# Supplementary material for: Spatial transition tensor of single cells
Source: Nat Methods. 2024 May 16;21(6):1053–62. doi: 10.1038/s41592-024-02266-x (PMC11166574; doi:10.1038/s41592-024-02266-x)
Supplement: Supplementary file 1 — Supplementary Notes 1 and 2, Tables 1–3 and Figs. 1–10. [file 41592_2024_2266_MOESM1_ESM.pdf]

nature methods

Article

https://doi.org/10.1038/s41592-024-02266-x

Spatial transition tensor of single cells

In the format provided by the authors and unedited

Supplementary Note 1: Theoretical foundation of STT

The input to the STT is the single-cell gene expression matrices of both spliced(S) and unspliced(U) counts (**Fig.1c**), and the cell annotations that serve as initial guess for cell state membership. Through an iterative scheme between parameter estimation and dynamics decomposition, STT constructs an attractor-wise velocity tensor named transition tensor of shape $\mathbb{R}^{N_C \times 2 \times K \times N_G}$, where N_C denotes number of cells, N_G number of genes and K number of attractors. Other quantities of tensor-based dynamics, including the memberships of cells in the attractors, transition probabilities, and transition paths, are subsequently obtained in this construction (**main text Methods**).

Theoretically, STT is motivated by the stochastic model of gene expression and splicing dynamics within individual cells expressed as

$$\begin{cases} dU_i = (f_i(t, S_1, .., S_{N_G}) - \beta_i U_i)dt + \sigma_i dW_{i,t}, \\ dS_i = (\beta_i U_i - \gamma_i S_i)dt + \sigma_i dZ_{i,t}, \end{cases} \quad (1)$$

where U_i and S_i are the unspliced and spiced counts for gene *i*. The nonlinear function $f_i(t, S_1, .., S_{N_G})$ models how other genes regulate the production rate of gene *i*. The system can possess multiple fixed points or attractors representing the different cell types. The gene expression function permits the bifurcation of system modelling cell-fate commitment. The parameters β_i represents the mRNA splicing rate and γ_i is the mRNA degradation rate. The independent Wiener process term $W_{i,t}$ and $Z_{i,t}$ represent the effect of stochastic noise in gene expression. The presence of stochasticity also drives the rare noise-induced cell state transitions among multi-stable attractors, which typically occurs at the time scale larger than splicing dynamics.

To better estimate the parameters, STT assumes that the majority of cells are located within the multiple attractor basins that correspond to the different biological cell types, with a small fraction of cells making transitions across the saddle points[1]. Around these steady-states, the unspliced mRNA production term can be expanded and approximated to its linear expansion, thus introducing the attractor-dependent mRNA transcription rate

(**main text Methods**). Such expansion allows robust estimation of transition tensors parameters (**main text Fig.1d**, **Methods**).

A key step in STT to connect local dynamics, encoded by the attractor-wise transition tensor, to the global dynamics in development, is the construction of a tensor-based cellular random walk, whose transition probability matrix can be expressed as

$$P^w = (1-w)P^I + wP^D \quad (2)$$

Here P^I is the cellular transition probability matrix defined using the inner-product kernel between averaged tensor over attractors and cell's unspliced/spliced counts, and P^D is the transition probability matrix defined using connectivity(similarity) between cells' gene expression (**main text Methods** and ref[2]).The underlying rationale and insights to use P^w and especially P^i can be drawn from following theorem in ref[3]:

Theorem. *Denote* $x = (u,s) \in \mathbb{R}^{2G}$ *and* $v(x) = (f(t,u,s) - \beta u, \beta u - \gamma s) \in \mathbb{R}^{2G}$ *is the ground truth drift term in Eqn.(1) approximated by attractor-averaged transition tensor in STT. Define* $k_\epsilon(x_i,x_j) = d_\epsilon(x_i,x_j)v^I(x_i,x_j)$ with gaussian kernel $d_\epsilon(x_i,x_j) = e^{-\frac{|x_i-x_j|^2}{\varepsilon}}$ and inner-product velocity kernel $v^I(x_i,x_j) = e^{v^T(x_i)(x_j-x_i)}$. *Next, use* $k_\epsilon(x_i,x_j)$ *to induce transition probability matrix* $P^I_{\epsilon,\alpha}$ *with elements* $p_{ij} = \frac{k_\epsilon^{(\alpha)}(x_i,x_j)}{\sum_j k_\epsilon^{(\alpha)}(x_i,x_j)}$, *where* $k_\epsilon^{(\alpha)}(x_i,x_j) = \frac{k_\epsilon(x_i,x_j)}{q_\epsilon^\alpha(x_i)q_\epsilon^\alpha(x_j)}$ *in which the power value* $\alpha \in [0,1]$ *and* $q_\epsilon(x) = \sum_y k_\epsilon(x,y)$. *Then as the cell number tends to infinity and bandwidth of Gaussian kernel* ϵ *tends to zero, the scaled infinitesimal generator of Markov Chain induced by* $P^I_{\epsilon,\alpha}$ *converges to that of stochastic differential equation*

$$dX_t = [v(x) + 2(1-\alpha)\nabla\phi(x)]dt + \sqrt{2}dW_t.$$

Where $\phi(x) = -\ln\mu(x)$ *and* $\mu(x)$ *is the density of data. Under appropriate conditions, the convergence of generator leads to the weak convergence of stochastic process*[4] *(i.e. convergence in probability distribution).*

In addition, if P^d in Eqn.(2) is purely induced by Gaussian kernel, then infinitesimal generator of P^w in Eqn.(2) converges to that of

$$dX_t = \left[-\left(w + 2(1-\alpha)\right)\nabla\phi(x) + w\ell(x)\right]dt + \sqrt{2}dW_t$$

Where $\ell(x) = v(x) + \nabla\phi(x)$ is the non-equilibrium part of ground truth drift term.

In the STT implementation, the gaussian kernel with bandwidth ϵ was replaced by k-nearest-neighbor graph adopted by the CellRank package, which yields the similar continuum limits as cell number tends to infinity[3]. Overall, the theorem suggest that transition tensors can provide a cellular random walk description that is asymptotically consistent with continuous stochastic differential equation by constructing inner-product velocity kernel.

In addition to the global dynamics induced by inner-product kernel, to visualize the local transition tensor components, STT plots the streamlines using the function in scVelo, which adopted the cosine similarity between tensor's direction and cell's displacement[5]. The choice could be supported by following theorem in ref[3] to guarantee the accuracy of streamlines in continuum limit as cell numbers go to infinity.

Theorem. *Define* $k_\epsilon(x_i, x_j) = d_\epsilon(x_i, x_j)v^C(x_i, x_j)$ with gaussian kernel $d_\epsilon(x_i, x_j) = e^{-\frac{\|x_i - x_j\|^2}{\varepsilon}}$ and inner-product velocity kernel $v^C(x_i, x_j) = e^{\cos\langle v(x_i), x_j - x_i\rangle}$. *Next, use $k_\epsilon(x_i, x_j)$ to induce transition probability matrix P_ϵ^C with elements $p_{ij} = \frac{k_\epsilon(x_i, x_j)}{q_\epsilon(x_i)}$, where $q_\epsilon(x) = \sum_y k_\epsilon(x, y)$. Then as the cell number tends to infinity and bandwidth of Gaussian kernel ϵ tends to zero, the scaled infinitesimal generator of Markov Chain induced by P_ϵ^C converges to that of deterministic differential equation following the streamlines of $v(x)$,*

$$\frac{dx}{dt} = \hat{v}(x) := \frac{v(x)}{\|v(x)\|}.$$

Supplementary Note 2: Details of simulation and data analysis

The key hyper-parameters involving STT computation were recorded in Supplementary Table 2 and the GitHub notebook link.

Synthetic dataset of toggle-switch circuit

We used the Euler-Maruyama method to simulate the stochastic differential equation model of circuit[6] containing two mutually inhibited genes X and Y to generate both unspliced and spliced counts:

$$\frac{dU_X}{dt} = k_X \frac{1}{1 + \left(\frac{S_Y}{Y_0}\right)^n} - \beta U_X \ + \sigma\sqrt{U_X}\xi_{U_X,t}$$

$$\frac{dU_Y}{dt} = k_Y \frac{1}{1 + \left(\frac{S_X}{X_0}\right)^n} - \beta U_Y + \sigma\sqrt{U_Y}\xi_{U_Y,t}$$

$$\frac{dS_X}{dt} = \beta U_X - \gamma S_X \ + \sigma\sqrt{S_X}\xi_{S_X,t}$$

$$\frac{dS_Y}{dt} = \beta U_Y - \gamma S_Y + \sigma\sqrt{S_Y}\xi_{S_Y,t}$$

Under appropriate parameter ranges, the system has two stable fixed points. In simulation, we set $n = 2, k_X = 2, k_Y = 1, X_0 = Y_0 = 1, \beta = 1, \gamma = 0.1$ and set $\sigma = 0.6$ to include sufficient transition cells. We simulated 5 trajectories starting around the first stable fixed point $y_1 = (1,0,10,0)$ and another 5 starting around the second stable fixed point $y_2 = (0,1,0,10)$, each with an initial condition perturbed by Gaussian noise, i.e. $x_0 = \left(1 + \mathcal{N}(0,0.01)\right) * y_1$ or $x_0 = \left(1 + \mathcal{N}(0,0.01)\right) * y_2$. The simulation was conducted in time range [0,200] with time step $dt = 0.1$. Together, the dataset with 10010 data points in $\mathbb{R}^4$ was generated.

To analyze the generated dataset, we used the result of Leiden clustering for both spliced and unspliced counts under resolution 0.002 as the initial guess for attractor membership, which resulted in $K = 2$ attractors as input to STT.

To visualize the results, the 2D embedding of PCA using both spliced and unspliced counts was utilized. The streamlines of various tensor components were plotted using “pl.velocity_embedding_stream” function in scVelo where the velocity graph (“tl.velocity_graph”) was calculated using the cosine similarity between the tensor components and corresponding displacements in k-NN smoothed counts.

<u>Synthetic dataset of EMT circuit</u>

We used the SDE model of modified EMT circuit of nine molecular species based on Tian et al. to include unspliced and spliced counts[6]. Noise terms were added following the same approach of the toggle-switch model. The deterministic system has two saddle-node bifurcations with the increase of extrinsic TGFB levels, where three stable states (E, ICS and M) could co-exist within a certain parameter range. In simulation, we increase the extrinsic TGFB values from 0.6 to 2.4 by step of 0.2, and for each value, we simulate the trajectory within the time interval [0,1000] with timestep size 1e-4 under noise level $\sigma = 0.01$. One in every 2000 time points of the trajectory was collected to generate a dataset. Altogether, we generated a dataset containing 5000 cells and unspliced/spliced counts of 9 genes under various extrinsic TGFB levels.

To analyze the generated dataset, we used the result of Leiden clustering for both spliced and unspliced counts under resolution 0.01 as the initial guess for attractor membership, which resulted in $K = 3$ attractors as input to STT.

To visualize the results, the 2D embedding of UMAP using both spliced and unspliced counts was utilized. The streamlines of various tensor components were plotted using “pl.velocity_embedding_stream” function in scVelo where the velocity graph (“tl.velocity_graph”) was calculated using the cosine similarity between the tensor components and corresponding displacements in k-NN smoothed counts.

<u>Human lung A549 EMT dataset</u>

The dataset was downloaded and processed as in ref[6] with 3132 cells and 2000 highly variable selected genes. We used the temporal label as the initial guess for attractor membership, and chosed in $K = 3$ attractors as input to STT.

Adult bone marrow dataset

The dataset was downloaded using the scv.datasets. bonemarrow () from the scVelo package, the counts were normalized and the top 2000 highly variable genes were selected. We used original annotation as the initial guess for attractor membership, which resulted in $K = 7$ attractors as input to STT.

To visualize the results, the 2D embeddings of transition coordinate of STT (**main text Methods**) were utilized. The streamlines of various tensor components were plotted using "pl.velocity_embedding_stream" function in scVelo where the velocity graph ("tl.velocity_graph") was calculated using the cosine similarity between the tensor components and corresponding displacements in k-NN smoothed counts.

Mouse brain spatial dataset

The processed data was downloaded from https://zenodo.org/record/6798659 and we followed the SIRV pipeline to impute the unspliced and spliced counts. For imputed data, all 117 genes are used for calculation. We used region annotation as the initial guess for attractor membership, which resulted in $K = 8$ attractors as input to STT.

To visualize the results, the spatial coordinates in original spatial data were utilized. The streamlines of various tensor components were plotted using "pl.velocity_embedding_stream" function in scVelo where the velocity graph ("tl.velocity_graph") was calculated using the cosine similarity between the tensor components and corresponding displacements in k-NN smoothed counts.

Chicken heart spatial dataset

The processed data was downloaded from https://zenodo.org/record/6798659 and we followed the SIRV pipeline to impute the unspliced and spliced counts. For imputed data,

top 2000 genes selected by scanpy.pp.highly_variable_genes are used for calculation, with 'flavor=seurat v3'. We used the region annotation as the initial guess for attractor membership, which resulted in $K = 5$ attractors as input to STT.

To visualize the results, the spatial coordinates in original spatial data were utilized. The streamlines of various tensor components were plotted using "pl.velocity_embedding_stream" function in scVelo where the velocity graph ("tl.velocity_graph") was calculated using the cosine similarity between the tensor components and corresponding displacements in k-NN smoothed counts.

Mouse coronal hemibrain spatial dataset

The processed data was downloaded from https://www.dropbox.com/s/c5tu4drxda01m0u/mousebrain_bin60.h5ad?dl=0. We used top 2000 genes selected by scanpy.pp.highly_variable_genes are used for calculation, with 'flavor=seurat v3'. We used the region annotation as the initial guess for attractor membership, which resulted in $K = 15$ attractors as input to STT.

To visualize the results, the spatial coordinates in original spatial data were utilized. The streamlines of various tensor components were plotted using "pl.velocity_embedding_stream" function in scVelo where the velocity graph ("tl.velocity_graph") was calculated using the cosine similarity between the tensor components and corresponding displacements in k-NN smoothed counts.

Supplementary Tables

Table S1 Key hyper-parameters in STT dynamical analysis function

Parameter	Meaning	Default
n_states	Number of attractors assigned in the STT analysis	Annotation; Clustering
n_neighbors	Number of nearest neighbors used when constructing the tesnor-induced kernel	0.01* cell numbers
weight_connectivities	Relative weight of cell gene-expression similarity-induced random walk kernel against tensor-induced kernel	0.5
spa_weight	Relative weight of spatial random walk aginst other kernels	0.3
n_components	Number of components used when estimating cell membership using GPCCA random walk transition probability matrix decomposition	21
l2	Regularization factor of parameters in tensor kinetic estimation	0.1
thresh_entropy	Threshold of the 75% quantile of the cell membership relative entropy differences with last iteration to terminate iteration	0.1
thresh_ms_gene	Threshold of minimum multi-stability score to select genes in the analysis in each iteration	0.2

Table S2 Summary table of hyper-parameters in datasets analyzed

Datasets	n_states	n_neighbors	weight_connectivities	spa_weight	n_components	L2	thresh_entropy	thresh_ms_gene
Toggle switch	2	100	0.2	n/a	20	0.1	0.1	0.2
EMT circuit	3	50	0.5	n/a	21	0.1	0.1	0.2
A549 EMT	3	100	0.5	n/a	21	0.1	0.1	0.2
Bone marrow	7	60	0.5	n/a	21	0.1	0.1	0
pancreas	8	25	0.5	n/a	21	0.1	0.1	0
Mouse brain (HybISS)	8	50	0.5	0.3	21	0.1	0.1	0.2
Chicken heart	5	20	0.5	0.3	21	0.1	0.1	0.2
Mouse brain (Stereo-seq)	15	70	0.5	0.3	21	0.1	0.1	0.2

Table S3 Summary table of STT computation time and memory usage when analyzing single-cell and spatial transcriptome datasets in the manuscript, tested on a personal laptop. Here peak memory indicates the maximum amount of total memory used during running, within which the incremental memory denotes the memory requested when executing the dynamical analysis API of STT. The memory usage was recorded by the Python memory profiler package. The timing was recorded by taking the average of 7 executions by the Python timeit package. The performance was tested on a MacBook Air 2023 with M2 chip and 16GB memory.

Datasets	Number of Cells	Number of Genes (input to algorithm)	Running Time (mean +/- std)	Peak Memory/Incremental
A549 EMT	3132	2000	125s± 13.5 s	2039.59 MB/1412.30 MB
Bone marrow	5780	2000	272s± 76 s	3004.10 MB/ 2759.29 MB
Pancreas	2531	2000	100s± 9.44 s	2088.36 MB/ 1573.52 MB
Mouse brain (HybISS)	4628	117	24 s ± 4.97s	847.57 MB/ 502.52 MB
Chicken heart	1967	2000	65s± 8.63 s	1563.92 MB/630.31 MB
Mouse brain (Stereo-seq)	7765	2000	387s± 52 s	5330.27 MB/4350.57 MB

Supplementary Figures

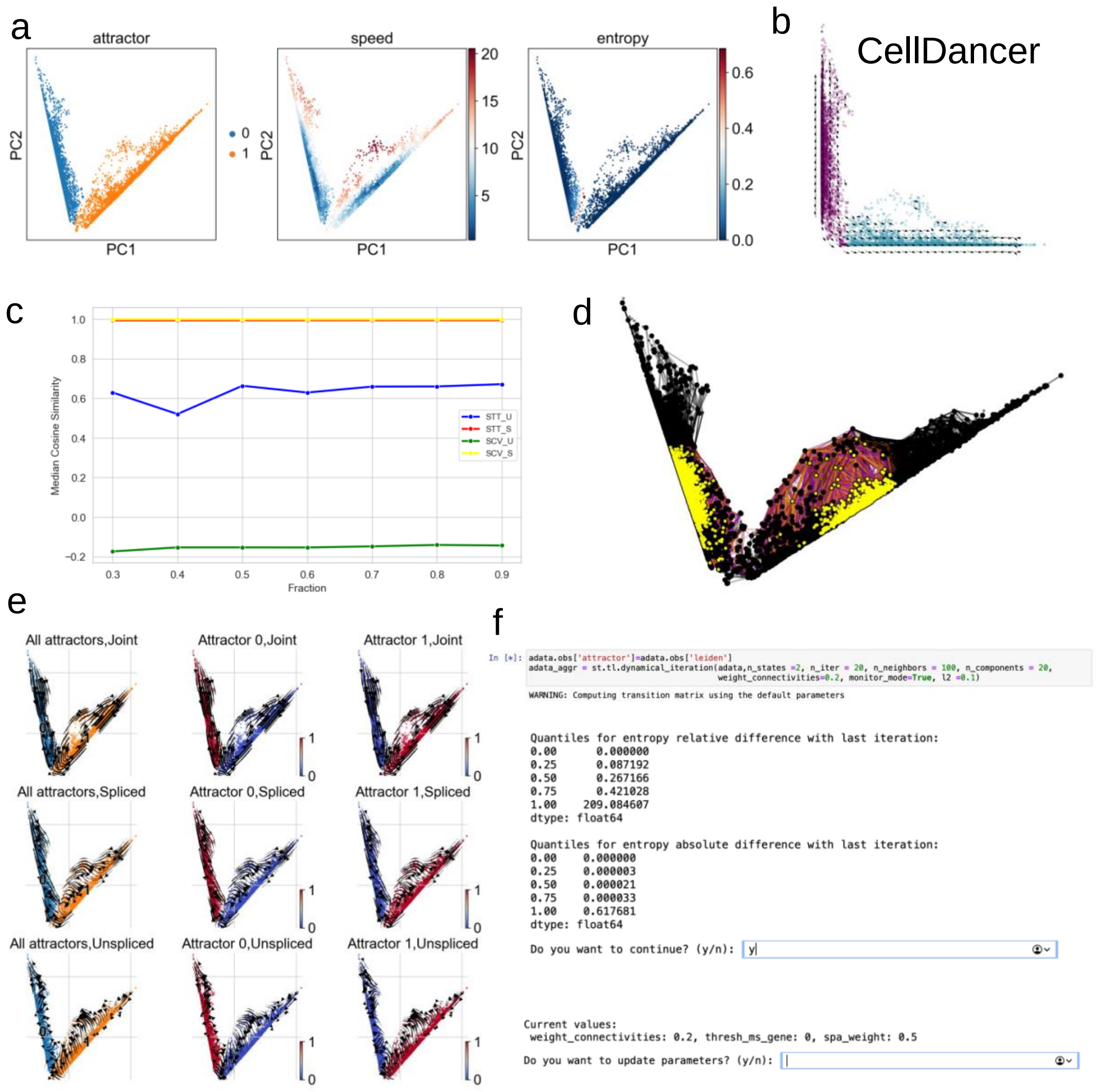

Figure S1. *Additional analysis result for toggle switch simulation dataset.* (a) Additional output of STT analysis on toggle switch data including attractor membership (left), speed (middle), and cell entropy (right). (b) Streamlines calculated by CellDancer. (c) Cosine similarity between the unspliced and spliced components of the inferred velocity (STT or scVelo) and ground-truth vector when sub-sampling the dataset. (d) Simulation of the random walk induced by STT multi-stability kernel using CellRank. The black points denote the starting points and yellow points denote the ending points of simulated random walks corresponding to attractors. (e) The tensor streamlines of various components (both, only spliced, only unspliced) when projected on either both attractors or a single attractor. (f)The interface of monitor module of STT iteration scheme.

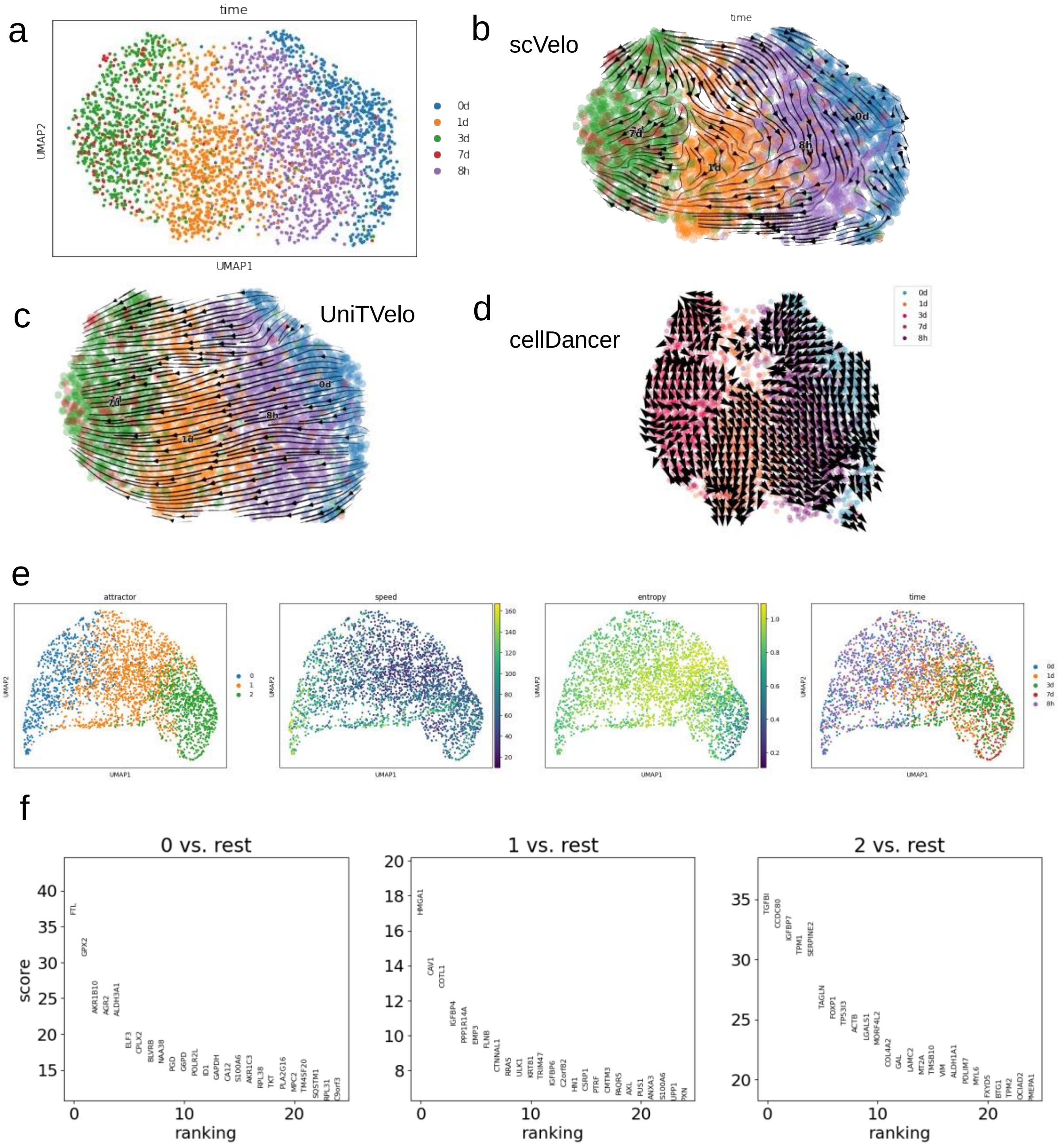

Figure S2 *Additional analysis result for A549 dataset.* (a) The UMAP embedding of spliced counts only with cells color-coded by collection time. (b-d) Streamlines of RNA velocity inferred by other methods. (e) Visualization of leiden clustering, averaged tensor speed, cellular entropy and collection time in UMAP of both unspliced and spliced counts (of 2000 highly variable genes). (f) The differentially expressed (DE) genes of each attractor identified by the Wilcox test using Scanpy.

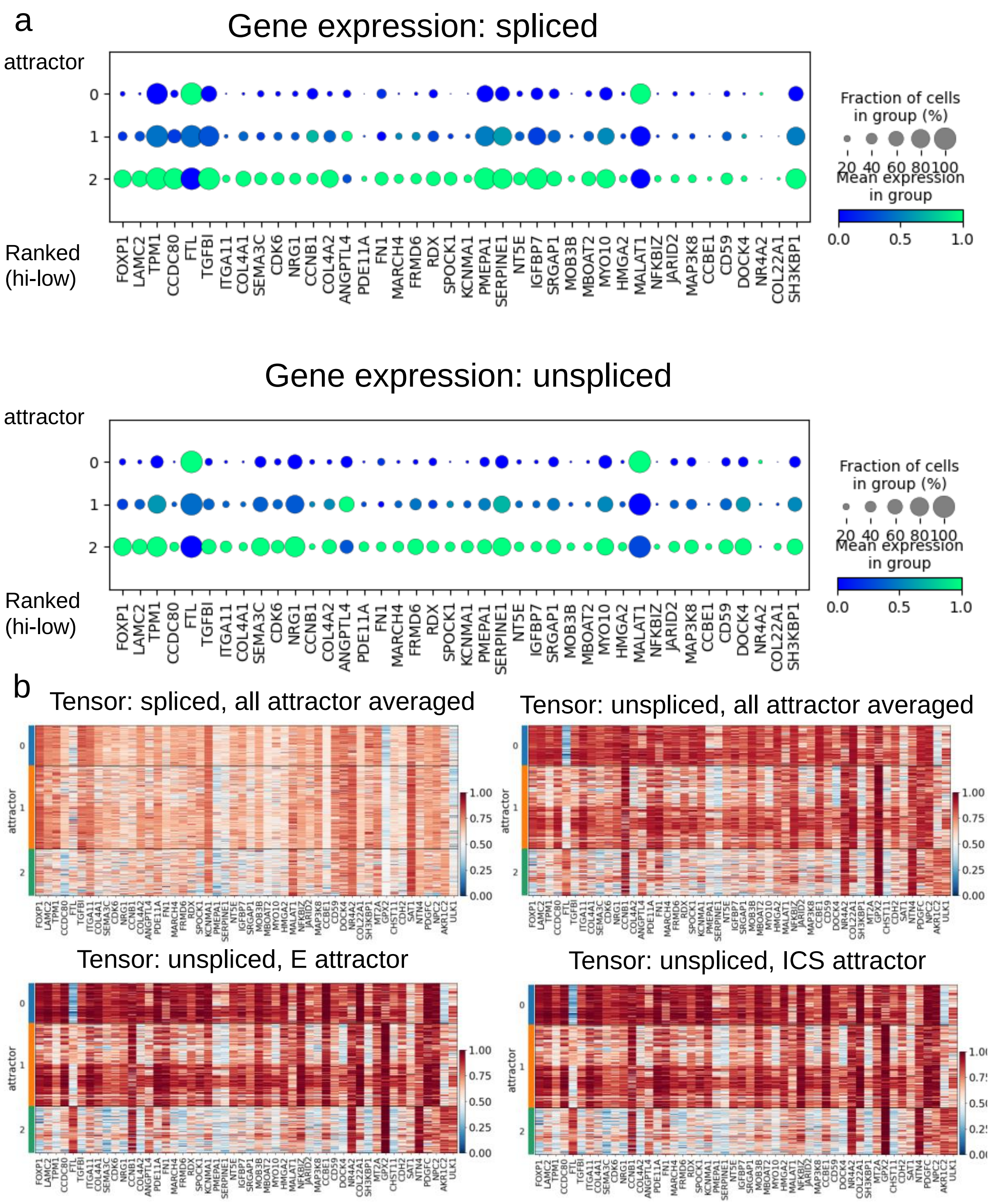

Figure S3 *Gene and tensor analysis for A549 dataset.* (a) The gene expression of top multi-stable genes among various attractors identified by STT for both spliced (top) and unspliced (bottom) RNA counts (0=Epithelial, 1=ICS, 2=Mesenchymal). (b) The heatmap of tensor components.

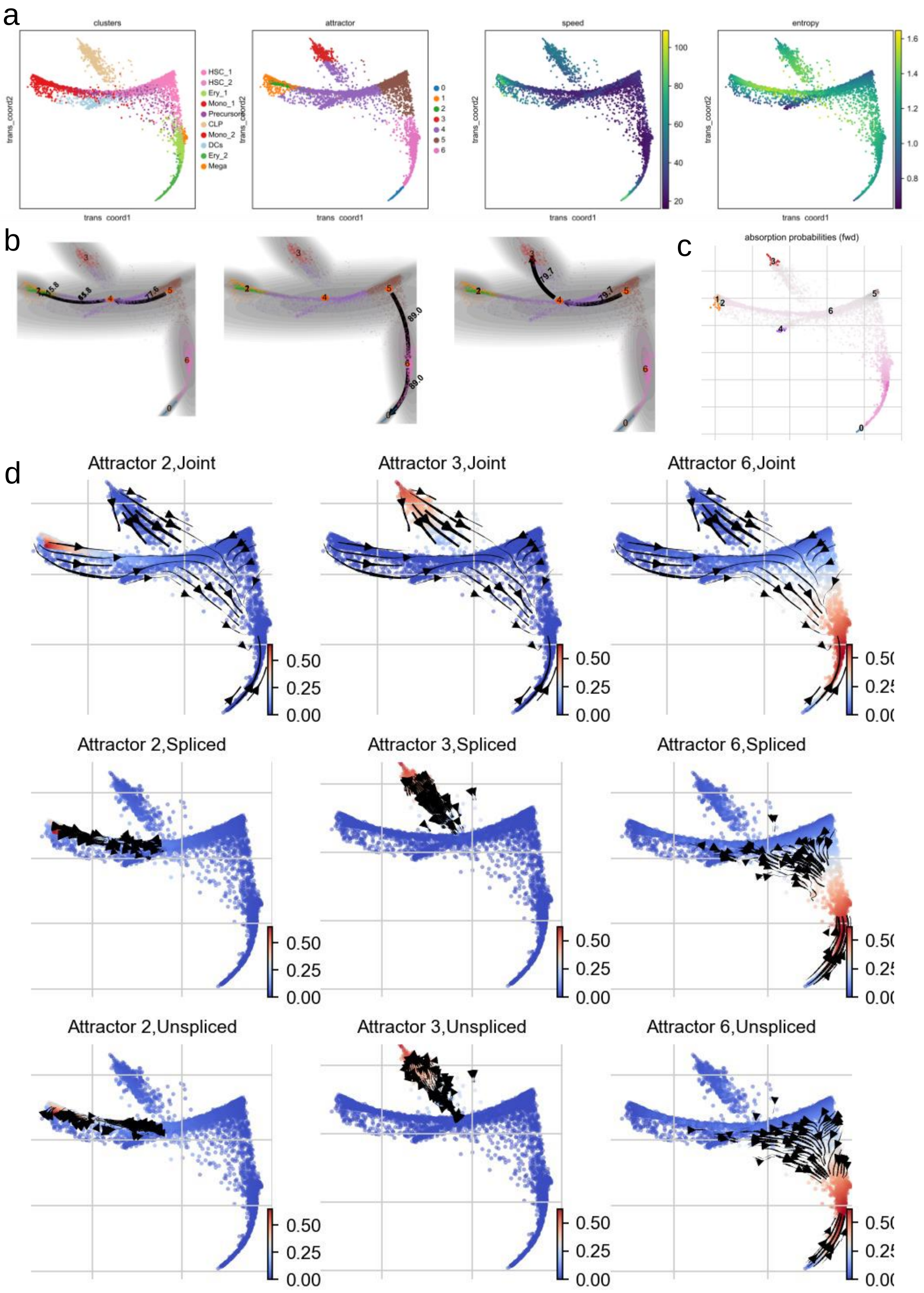
a
clusters
attractor
speed
entropy
trans_coord1
trans_coord2
HSC_1
HSC_2
Ery_1
Mono_1
Precursors
CLP
Mono_2
DCs
Ery_2
Mega
b
c
absorption probabilities (fwd)
d
Attractor 2,Joint
Attractor 3,Joint
Attractor 6,Joint
Attractor 2,Spliced
Attractor 3,Spliced
Attractor 6,Spliced
Attractor 2,Unspliced
Attractor 3,Unspliced
Attractor 6,Unspliced
0.50
0.25
0.00

Figure S4. *Analysis result for human bone marrow blood development dataset.* (a) Output quantities of STT including (from left to right): cell annotations, attractor membership, tensor speed and cellular entropy. (b) Transition paths calculated from HSC to multiple cell fates. The cells are embedded in the transition coordinates of constructed dynamical manifold. (c) The CellRank absorption probability analysis based on STT multi-stability kernel. (d) The streamlines of various tensor components (both, only spliced, only unspliced) when projected on attractors 2, 3, and 6.

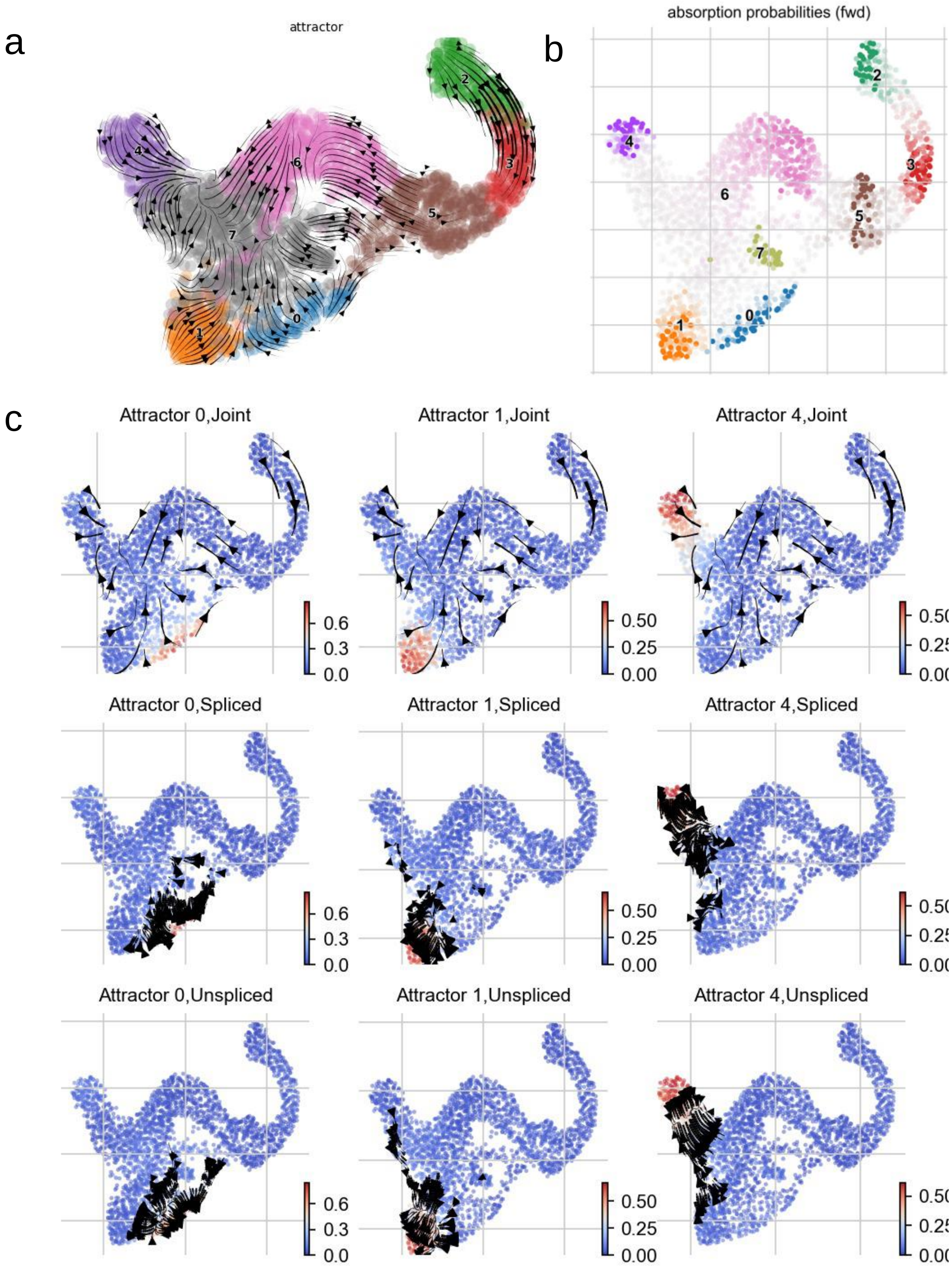

Figure S5. *Analysis result for pancreas dataset.* (a) Attractors predicted by STT. (b) The CellRank absorption probability analysis based on STT multi-stability kernel. (c) The streamlines of various tensor components.

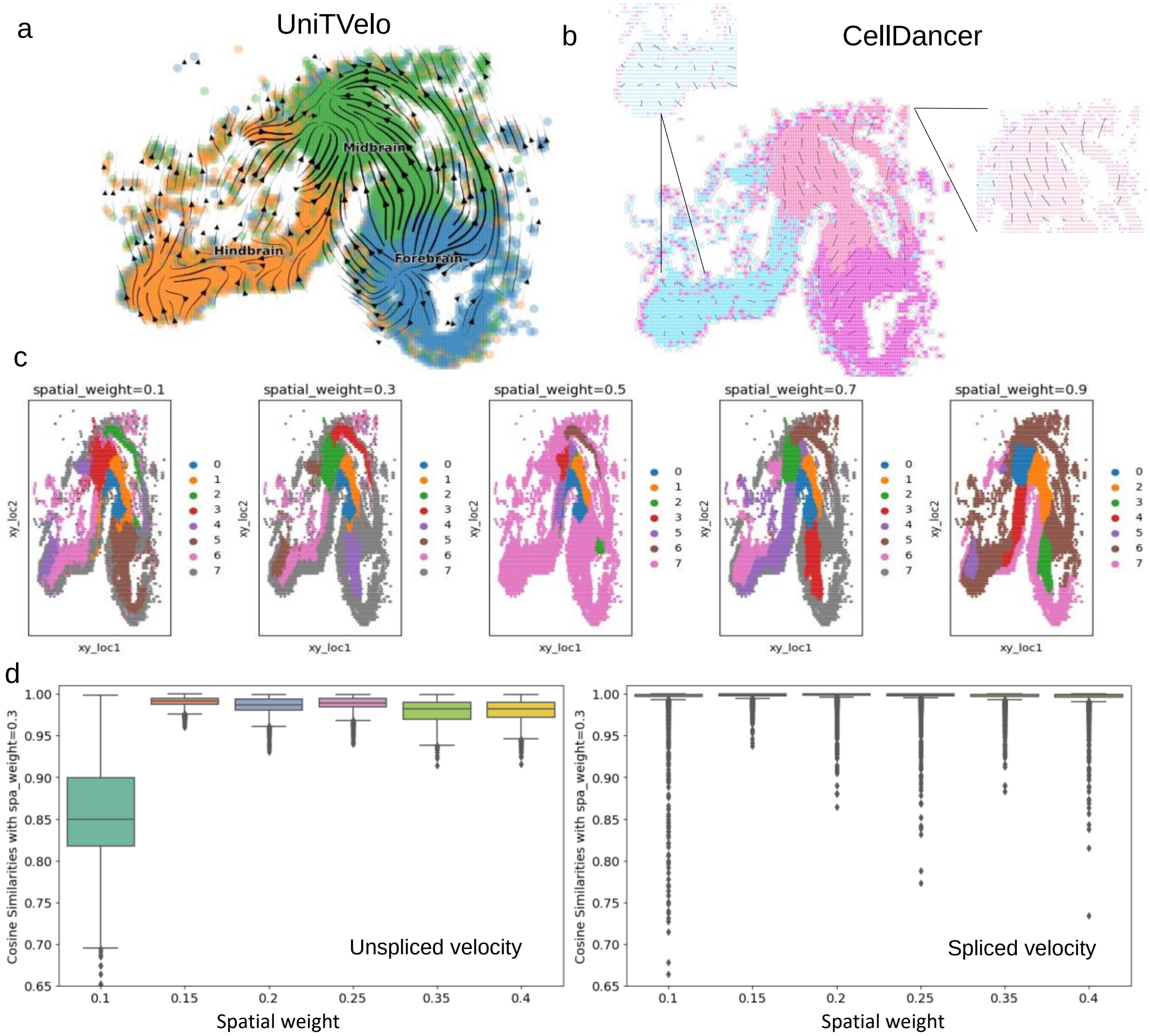

Figure S6 *Additional analysis for mouse brain spatial dataset.* (a-b) Streamlines of RNA velocity calculated by UniTVelo (a) and CellDancer (b). (c) The STT attractors detected under increasing values of the spatial weight parameter from 0.1 to 0.9. (d) Parameter sensitivity analysis of spatial weight kernel. Under each spatial weight, for each cell we calculated the cosine similarity between its attractor-averaged tensor (unspliced or spliced component) with the default case when spatial weight was assigned as 0.3. The central box represents the interquartile range (IQR), from the 25th (bottom bounds) to 75th percentiles (top bounds) and horizontal line within the box indicates the median (50th percentile). The whiskers stretch out to the values that fall within 1.5 times the IQR from the lower and upper quartiles. The dots indicate outliers.

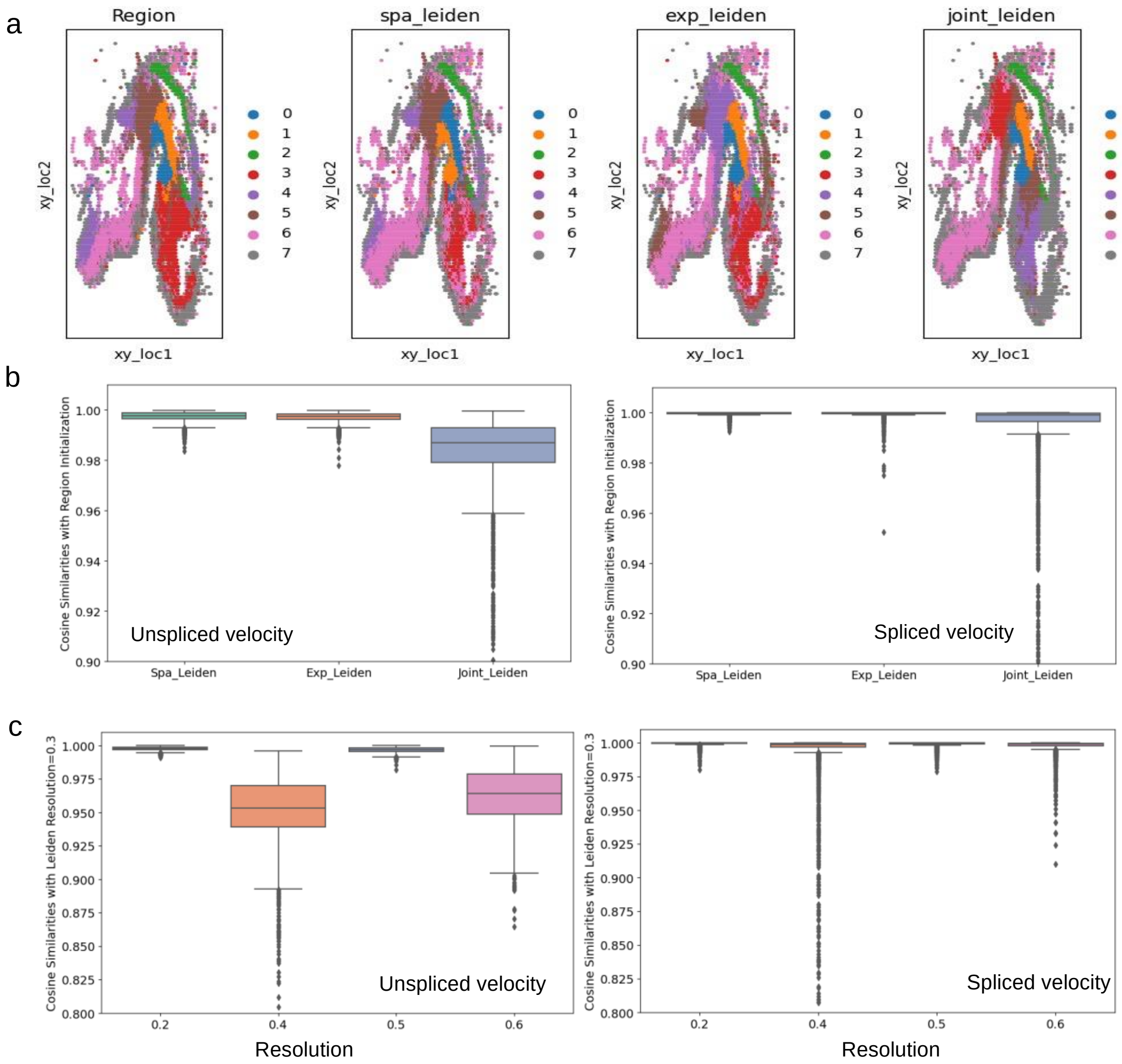

Figure S7 *Sensitivity analysis for iteration initiation of mouse brain spatial dataset.* (a) The attractors detected under different cell assignment initialization strategies. Here "Region" employs the original annotation in dataset about spatial regions, "spa_leiden" employs the leiden clustering of the spatial coordinates, "exp_leiden" employs the leiden clustering of the gene expression counts, and "joint_leiden" employs the leiden clustering of both spliced and unspliced counts. (b) Consistency of tensor results under various initialization strategies. Under each strategy, for each cell we calculated the cosine similarity between its attractor-averaged tensor (unspliced or spliced component) with the case applied in the main text where the region annotation was used as default. (c) Parameter sensitivity analysis of Leiden clustering resolution for STT initialization. Under each resolution, for each cell we calculated the cosine similarity between its attractor-averaged tensor (unspliced or spliced component) with the case applied in "exp_leiden" of (b), where the resolution was assigned as 0.3. The central box represents the

interquartile range (IQR), from the 25th (bottom bounds) to 75th percentiles (top bounds) and horizontal line within the box indicates the median (50th percentile). The whiskers stretch out to the values that fall within 1.5 times the IQR from the lower and upper quartiles. The dots indicate outliers.

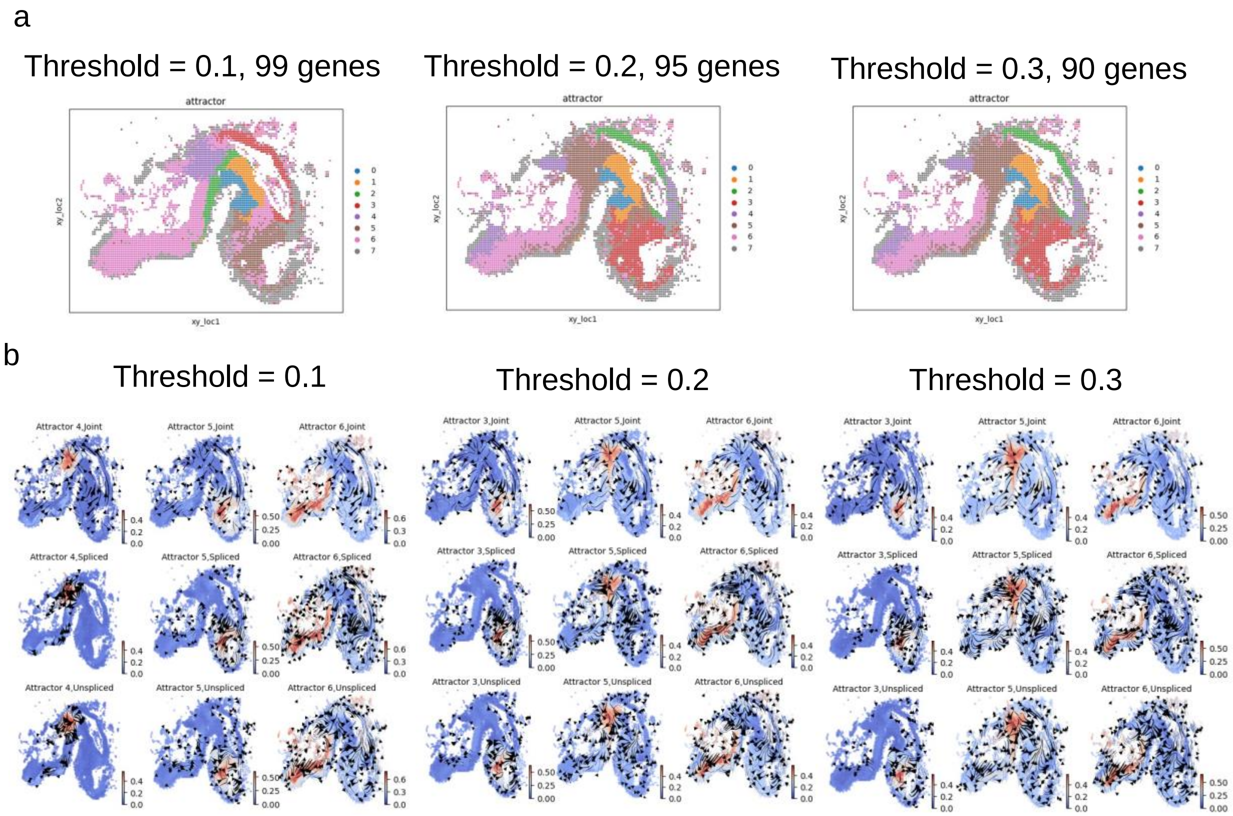

Figure S8 *Sensitivity analysis of threshold to filter multi-stability genes in mouse brain spatial dataset.* (a) The STT attractors detected for increasing values of multi-stability genes score from 0.1 to 0.3. (b) Corresponding streamlines of tensor components.

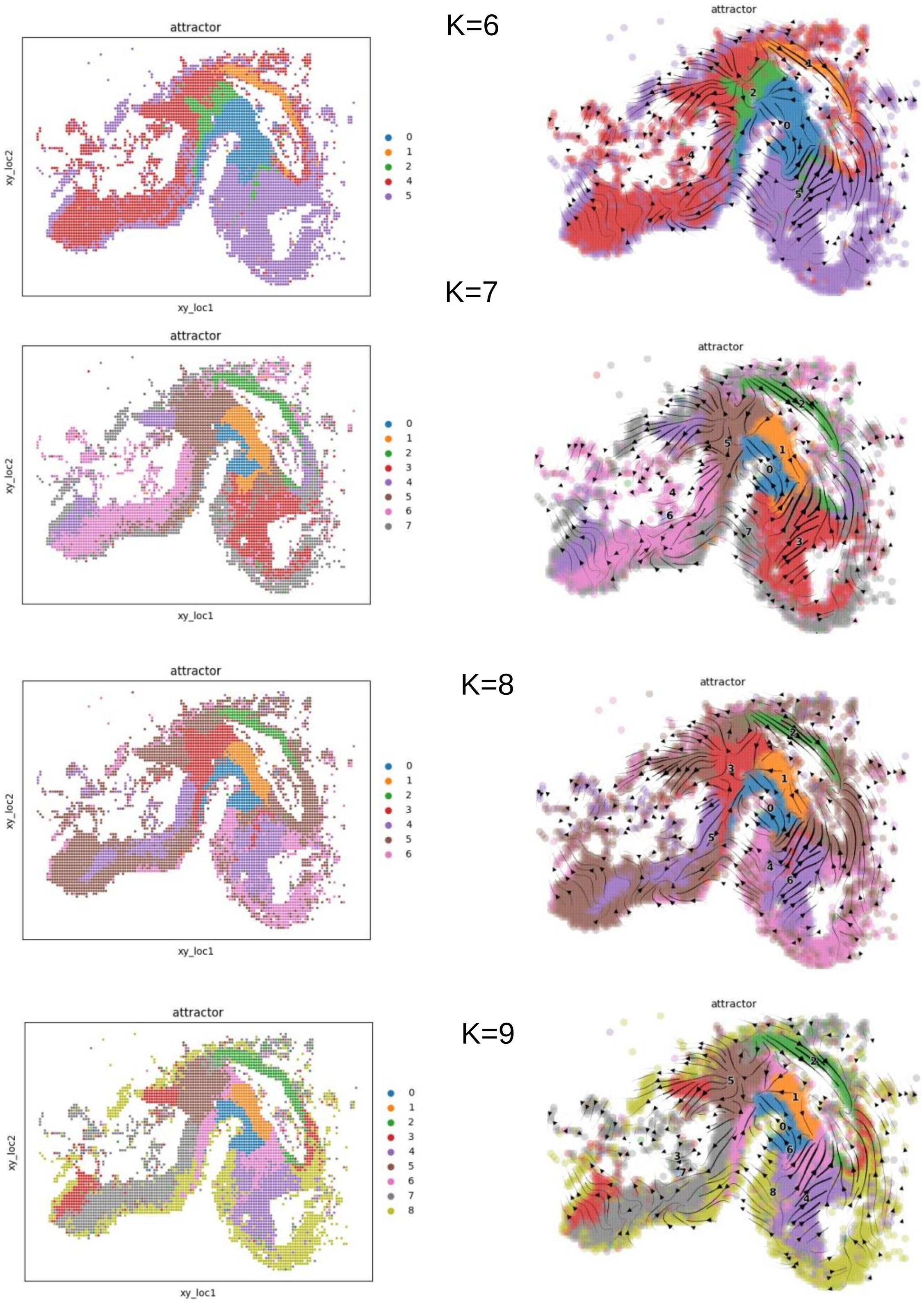

Figure S9 *Sensitivity analysis of number of clusters in mouse brain spatial dataset.* Left: Attractors detected by STT. Right: Corresponding streamlines of averaged tensor across all attractors.

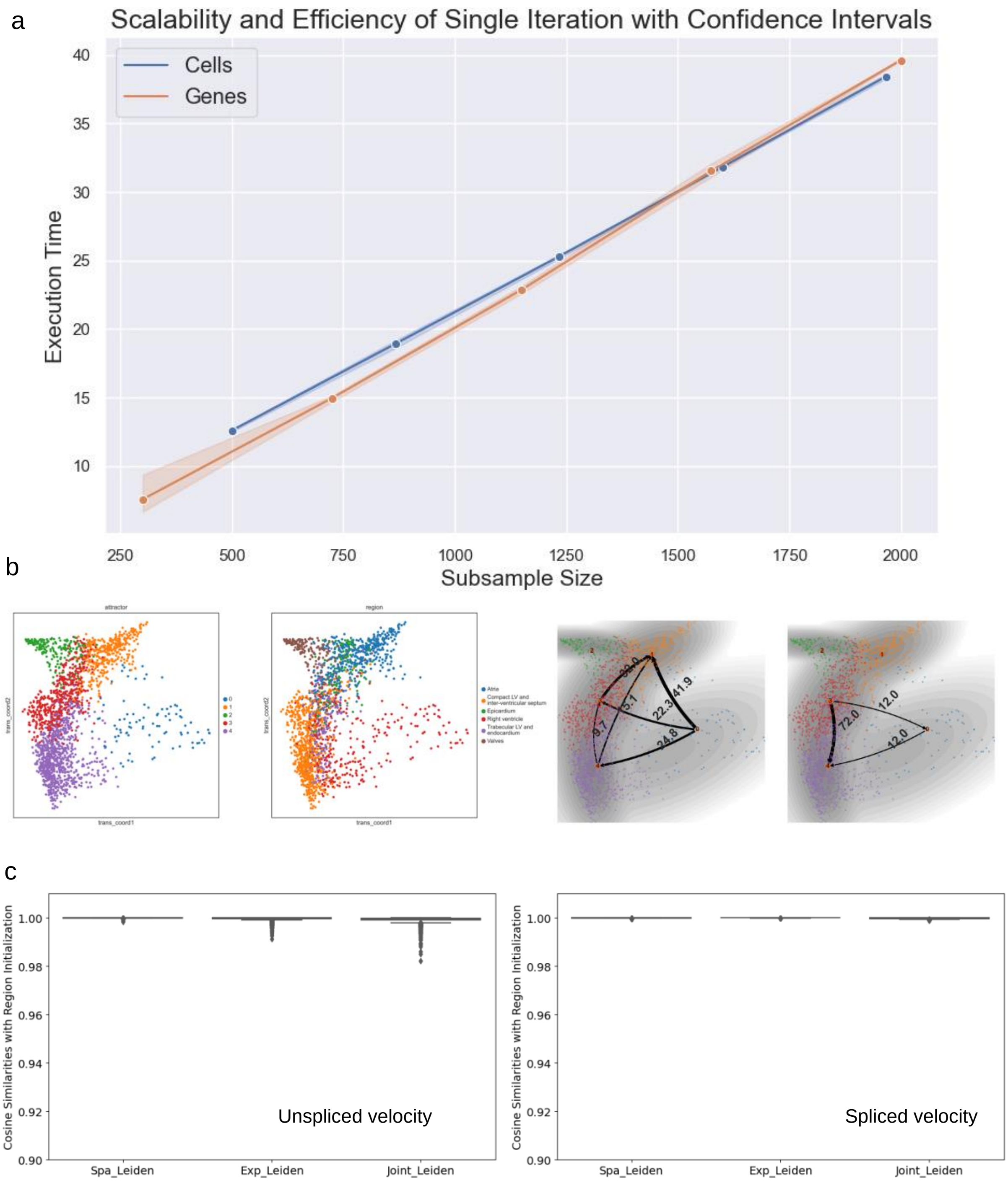

Figure S10 *Additional analysis on chicken heart spatial dataset.* (a) Test of scalability of algorithm when subsampling genes or cells. Shaded area indicates standard deviation of execution time over multiple runs (n=3). (b) The transition coordinate embedding of cells and their inferred transition paths. (c) Sensitivity analysis STT initialization strategy tested on chicken heart 10X Visium dataset. Under each strategy, for each cell we calculated the cosine similarity between its attractor-averaged tensor (unspliced or spliced component) with the case applied in the main text where the region annotation was used

as default. The central box represents the interquartile range (IQR), from the 25th (bottom bounds) to 75th percentiles (top bounds) and horizontal line within the box indicates the median (50th percentile). The whiskers stretch out to the values that fall within 1.5 times the IQR from the lower and upper quartiles. The dots indicate outliers.

References

1. Zhou, P., et al., *Dissecting transition cells from single-cell transcriptome data through multiscale stochastic dynamics.* Nature Communications, 2021. **12**(1): p. 5609.
2. Lange, M., et al., *Cellrank for directed single-cell fate mapping.* Nature methods, 2022. **19**(2): p. 159.
3. Li, T., et al., *On the mathematics of rna velocity i: Theoretical analysis.* CSIAM Transactions on Applied Mathematics, 2021. **2**(1): p. 1.
4. Ethier, S.N., et al., *Markov processes: Characterization and convergence*. 2009: John Wiley & Sons.
5. Bergen, V., et al., *Generalizing rna velocity to transient cell states through dynamical modeling.* Nat Biotechnol, 2020. **38**(12): p. 1408.
6. Bocci, F., et al., *Splicejac: Transition genes and state‐specific gene regulation from single‐cell transcriptome data.* Molecular Systems Biology, 2022. **18**(11): p. e11176.
