# Supplementary material for: Spatial transition tensor of single cells
Source: Nat Methods. 2024 May 16;21(6):1053–62. doi: 10.1038/s41592-024-02266-x (PMC11166574; doi:10.1038/s41592-024-02266-x)
Supplement: Supplementary file 2 — Reporting Summary [file 41592_2024_2266_MOESM2_ESM.pdf]

nature portfolio

Corresponding author(s): Qing Nie

Last updated by author(s): Feb 7, 2024

Reporting Summary

Nature Portfolio wishes to improve the reproducibility of the work that we publish. This form provides structure for consistency and transparency in reporting. For further information on Nature Portfolio policies, see our Editorial Policies and the Editorial Policy Checklist.

Statistics

For all statistical analyses, confirm that the following items are present in the figure legend, table legend, main text, or Methods section.

n/a	Confirmed	
☐	☒	The exact sample size (*n*) for each experimental group/condition, given as a discrete number and unit of measurement
☐	☒	A statement on whether measurements were taken from distinct samples or whether the same sample was measured repeatedly
☐	☒	The statistical test(s) used AND whether they are one- or two-sided<br>*Only common tests should be described solely by name; describe more complex techniques in the Methods section.*
☒	☐	A description of all covariates tested
☒	☐	A description of any assumptions or corrections, such as tests of normality and adjustment for multiple comparisons
☒	☐	A full description of the statistical parameters including central tendency (e.g. means) or other basic estimates (e.g. regression coefficient) AND variation (e.g. standard deviation) or associated estimates of uncertainty (e.g. confidence intervals)
☒	☐	For null hypothesis testing, the test statistic (e.g. *F*, *t*, *r*) with confidence intervals, effect sizes, degrees of freedom and *P* value noted<br>*Give P values as exact values whenever suitable.*
☒	☐	For Bayesian analysis, information on the choice of priors and Markov chain Monte Carlo settings
☒	☐	For hierarchical and complex designs, identification of the appropriate level for tests and full reporting of outcomes
☒	☐	Estimates of effect sizes (e.g. Cohen's *d*, Pearson's *r*), indicating how they were calculated

Our web collection on statistics for biologists contains articles on many of the points above.

Software and code

Policy information about availability of computer code

Data collection	The bone marrow dataset and pancreas dataset was downloaded using cellrank (1.3.1) and scvelo (0.2.4) package. The mouse brain and checken heart data was provided with package SIRV 1.0 (https://github.com/tabdelaal/SIRV) with datasets deposited on zenodo with doi https://doi.org/10.5281/zenodo.6798659. The KEGG database was downloaded using gseapy==1.0.4
Data analysis	The STT software is available at and its dependency on other packages was described in the file. The software described in manuscript includes cellrank(1.3.1)which implements GPCCA algorithm, and pyEMMA(2.5.6) which implements transition paths analysis.

For manuscripts utilizing custom algorithms or software that are central to the research but not yet described in published literature, software must be made available to editors and reviewers. We strongly encourage code deposition in a community repository (e.g. GitHub). See the Nature Portfolio guidelines for submitting code & software for further information.

Data

Policy information about availability of data

All manuscripts must include a data availability statement. This statement should provide the following information, where applicable:

- Accession codes, unique identifiers, or web links for publicly available datasets
- A description of any restrictions on data availability
- For clinical datasets or third party data, please ensure that the statement adheres to our policy

All the datasets used in this paper are publicly available. The detailed preprocessing of datasets is described in Supplementary Note. The simulation datasets of

April 2023

synthetic circuits are available at https://github.com/cliffzhou92/STT/tree/release/data. The EMT dataset of human lung A549 cell lines is available at GSE147405. The pancreas dataset (originally available at GSE132188) and adult human bone marrow datasets (originally available at https://data.humancellatlas.org/explore/projects/091cf39b-01bc-42e5-9437-f419a66c8a45) are downloaded from the built-in datasets of scvelo==0.2.4 package (https://scvelo.readthedocs.io/api/#datasets). The spatial datasets of mouse brain and chicken heart as well as scRNA-seq datasets used for imputation are downloaded from https://zenodo.org/record/6798659. The stereo-seq mouse brain dataset with unspliced/spliced counts was downloaded from Spateo package(https://github.com/aristoteleo/spateo-tutorials, https://www.dropbox.com/s/c5tu4drxda01m0u/mousebrain_bin60.h5ad?dl=0). The KEGG database was originally available on Enrichr webpage (https://maayanlab.cloud/Enrichr/#librariesdownloaded) and downloaded using gseapy==1.0.4.

Research involving human participants, their data, or biological material

Policy information about studies with human participants or human data. See also policy information about sex, gender (identity/presentation), and sexual orientation and race, ethnicity and racism.

Reporting on sex and gender	n/a, this study does not involve human participants.
Reporting on race, ethnicity, or other socially relevant groupings	n/a, this study does not involve human participants.
Population characteristics	n/a, this study does not involve human participants.
Recruitment	n/a, this study does not involve human participants.
Ethics oversight	n/a, this study does not involve human participants.

Note that full information on the approval of the study protocol must also be provided in the manuscript.

Field-specific reporting

Please select the one below that is the best fit for your research. If you are not sure, read the appropriate sections before making your selection.

☒ Life sciences ☐ Behavioural & social sciences ☐ Ecological, evolutionary & environmental sciences

For a reference copy of the document with all sections, see nature.com/documents/nr-reporting-summary-flat.pdf

Life sciences study design

All studies must disclose on these points even when the disclosure is negative.

Sample size	To evaluate the performance of STT under various scenarios, we tested on two simulation datasets, three scRNA-seq datasets and three spatial transcriptome datasets sequenced by different technology
Data exclusions	No data was excluded
Replication	We run the analysis code multiple times to ensure replication of results
Randomization	Randomization is not relevant to our study since we do not perform group allocation and comparison
Blinding	Blinding is not relevant to this study since we do not perform group allocation and comparison

Reporting for specific materials, systems and methods

We require information from authors about some types of materials, experimental systems and methods used in many studies. Here, indicate whether each material, system or method listed is relevant to your study. If you are not sure if a list item applies to your research, read the appropriate section before selecting a response.

Materials & experimental systems

n/a	Involved in the study
☒	☐ Antibodies
☒	☐ Eukaryotic cell lines
☒	☐ Palaeontology and archaeology
☒	☐ Animals and other organisms
☒	☐ Clinical data
☒	☐ Dual use research of concern
☒	☐ Plants

Methods

n/a	Involved in the study
☒	☐ ChIP-seq
☒	☐ Flow cytometry
☒	☐ MRI-based neuroimaging

Plants

Seed stocks

Report on the source of all seed stocks or other plant material used. If applicable, state the seed stock centre and catalogue number. If plant specimens were collected from the field, describe the collection location, date and sampling procedures.

Novel plant genotypes

Describe the methods by which all novel plant genotypes were produced. This includes those generated by transgenic approaches, gene editing, chemical/radiation-based mutagenesis and hybridization. For transgenic lines, describe the transformation method, the number of independent lines analyzed and the generation upon which experiments were performed. For gene-edited lines, describe the editor used, the endogenous sequence targeted for editing, the targeting guide RNA sequence (if applicable) and how the editor was applied.

Authentication

Describe any authentication procedures for each seed stock used or novel genotype generated. Describe any experiments used to assess the effect of a mutation and, where applicable, how potential secondary effects (e.g. second site T-DNA insertions, mosiacism, off-target gene editing) were examined.
